# Risk Profiling of Hookworm Infection and Intensity in Southern Lao People’s Democratic Republic Using Bayesian Models

**DOI:** 10.1371/journal.pntd.0003486

**Published:** 2015-03-30

**Authors:** Armelle Forrer, Penelope Vounatsou, Somphou Sayasone, Youthanavanh Vonghachack, Dalouny Bouakhasith, Jürg Utzinger, Kongsap Akkhavong, Peter Odermatt

**Affiliations:** 1 Department of Epidemiology and Public Health, Swiss Tropical and Public Health Institute, Basel, Switzerland; 2 University of Basel, Basel, Switzerland; 3 National Institute of Public Health, Ministry of Health, Vientiane, Lao People’s Democratic Republic; 4 Faculty of Basic Sciences, University of Health Sciences, Vientiane, Lao People’s Democratic Republic; Jiangsu Institute of Parasitic Diseases, CHINA

## Abstract

**Background:**

Among the common soil-transmitted helminth infections, hookworm causes the highest burden. Previous research in the southern part of Lao People’s Democratic Republic (Lao PDR) revealed high prevalence rates of hookworm infection. The purpose of this study was to predict the spatial distribution of hookworm infection and intensity, and to investigate risk factors in the Champasack province, southern Lao PDR.

**Methodology:**

A cross-sectional parasitological and questionnaire survey was conducted in 51 villages. Data on demography, socioeconomic status, water, sanitation, and behavior were combined with remotely sensed environmental data. Bayesian mixed effects logistic and negative binomial models were utilized to investigate risk factors and spatial distribution of hookworm infection and intensity, and to make predictions for non-surveyed locations.

**Principal Findings:**

A total of 3,371 individuals were examined with duplicate Kato-Katz thick smears and revealed a hookworm prevalence of 48.8%. Most infections (91.7%) were of light intensity (1-1,999 eggs/g of stool). Lower hookworm infection levels were associated with higher socioeconomic status. The lowest infection levels were found in preschool-aged children. Overall, females were at lower risk of infection, but women aged 50 years and above harbored the heaviest hookworm infection intensities. Hookworm was widespread in Champasack province with little evidence for spatial clustering. Infection risk was somewhat lower in the lowlands, mostly along the western bank of the Mekong River, while infection intensity was homogeneous across the Champasack province.

**Conclusions/Significance:**

Hookworm transmission seems to occur within, rather than between villages in Champasack province. We present spatial risk maps of hookworm infection and intensity, which suggest that control efforts should be intensified in the Champasack province, particularly in mountainous areas.

## Author Summary

Hookworm is a parasitic worm that can infect the human intestine. Infections are common in low- and middle-income tropical and subtropical countries, particularly where sanitation conditions are poor. Control programs aim at preventing heavy infection intensities, which are associated with morbidity. The aim of this study was to investigate risk factors for hookworm infection and intensity, and to predict their distribution in the Champasack province, southern Lao People’s Democratic Republic. We found that almost half of the 3,371 people examined were infected with hookworms. Better-off people had a lower risk and intensity of infection. Females showed lower prevalence and intensity of hookworm infection than males, except women aged 50 years and above, who were the most heavily infected. Preschool-aged children had the lowest infection levels. Hookworm transmission appeared to occur within, rather than between villages. Hookworm infections are widespread in the Champasack province, with homogeneous infection intensity and somewhat lower infection risk west of the Mekong River, which offers higher living conditions. Hookworm control should be intensified in the Champasack province, particularly in the mountainous areas.

## Introduction

Hookworm disease is caused by chronic infection with *Ancylostoma duodenale* or *Necator americanus* and is of considerable public health importance in low- and middle- income countries in the tropics and subtropics [1]. In 2010, an estimated 439 million people were infected with hookworm, causing a global burden of 3.2 million disability-adjusted life years [2,3]. Depending on hookworm infection intensity, the morbidity may range from mild and transient symptoms to severe disease, negatively impacting on child growth and cognitive development, and worker productivity in older age [4]. Chronic hookworm infection cause intestinal blood loss and may result in poor iron status and iron deficiency anemia, particularly in children, women of reproductive age, and individuals with high worm loads [4–6].

Preventive chemotherapy—that is the periodic administration of albendazole or mebendazole to school-aged children and other high-risk groups—is the backbone of the global control against hookworm and other soil-transmitted helminth infections [7,8]. The Ministry of Health (MoH) of Lao People’s Democratic Republic (Lao PDR) has adopted the national scheme for school deworming. Since 2005, two annual rounds of deworming are conducted among school-aged children [9]. In addition, anthelmintic treatment has been provided to preschool-aged children through the Expanded Program on Immunization (EPI), and alongside vitamin A distribution campaigns [10,11]. However, in face of continued exposure to contaminated environment and unhygienic behavior, re-infection with soil-transmitted helminths is rapid [12,13]. Hence, the identification and geographic delineation of areas that are at high risk of hookworm transmission is crucial for spatial targeting and fostering control activities.

The purpose of this study was to identify risk factors for hookworm infection and intensity in Champasack province, southern Lao PDR, and to predict infection risk and intensity in non-surveyed locations. These findings are important to enhance local control efforts and to inform the national helminthiasis control program.

## Methods

### Ethics Statement

The study was approved by the institutional research commission of the Swiss Tropical and Public Health Institute (Swiss TPH; Basel, Switzerland). Ethical approval was obtained from the MoH of Lao PDR (reference no. 027/NECHR) and the ethics committee of Basel (EKBB; reference no. 255/06). Permission for field work was obtained from the MoH, the Provincial Health Office (PHO), and the District Health Office (DHO). Because literacy is low in Champasack province, individual oral consent was obtained from all adult household members. Additionally, written informed consent was obtained from heads of households. This consent procedure was approved by the aforementioned ethics committees. Participants infected with hookworm were treated with a single 400 mg oral dose of albendazole. Other parasitic infections were treated according to national guidelines [14].

### Study Area

The study was carried out in Champasack, the largest province of southern Lao PDR, with an area of 15,415 km^2^ and a population of 603,370 in 2005 [15]. The province stretches from 13°55' to 15°29' N latitude and from 105°11' to 106°46' E longitude and is crossed by the Mekong River from North to South. The climate is of monsoon tropical type and the rainy season occurs between May and October. Soil-transmitted helminths, *Opisthorchis viverrini*, *Schistosoma mekongi*, and minute intestinal flukes (MIF) are endemic in this province [16–22].

### Parasitological, Demographic, Socioeconomic, and Behavioral Data

Epidemiological data were obtained from a cross-sectional, community-based survey carried out between January and May 2007 in all nine rural districts of Champasack province. The tenth district, which is primarily urban, was excluded. Sample selection was achieved using a two-stage sampling method: first a random selection of villages and, second, random selection of 10–15 households per village. All individuals aged 6 months and above were eligible. Overall, 4,380 participants in 51 villages were selected. A single stool sample was collected from each participant. Samples were screened for eggs of soil-transmitted helminths, *O. viverrini*, and *S. mekongi*. Duplicate 41.7 mg Kato-Katz thick smears were prepared from each stool sample the same day of collection, and the slides were examined under a microscope within 30–45 min by experienced laboratory technicians [23]. Helminth eggs were counted and recorded for each species separately. A random sample of 10% of the Kato-Katz thick smears were re-examined by a senior technician for quality control.

Data on demography (age, sex, ethnic group, main occupation, and educational attainment) and hygiene (hand washing, wearing shoes) were obtained from each participant by means of a pre-tested questionnaire. Heads of household were interviewed, and information collected about household characteristics, water and sanitation, and asset ownership. The geographic coordinates of each household were recorded using a hand-held global positioning system (GPS) device (Garmin Ltd.; Olathe, United States of America).

### Environmental Data

Enhanced vegetation index (EVI), day and night land surface temperature (LST), and land use/land cover (LULC) consisting of 18 land cover type 1 classes (IGBP) at a spatial resolution of 1 x 1 km were downloaded from Moderate Resolution Imaging Spectroradiometer (MODIS). Instead of normalized difference vegetation index (NDVI), EVI was used, since it is more sensitive to differences in heavily vegetated areas, and is more appropriate in areas such as Southeast Asia (see; http://earthobservatory.nasa.gov/Features/MeasuringVegetation/measuring_vegetation_4.php). Rainfall estimates (RFE) at 0.1 degree (about 10 x 11 km) resolution were obtained from the National Oceanic and Atmospheric Administration’s (NOAA) Climate Prediction Center (CPC) Famine Early Warning System (FEWS) Rainfall Estimates South Asia, version 2.0 (see; http://www.cpc.ncep.noaa.gov/products/fews/SASIA/rfe.shtml). Digital elevation data at a resolution of 90 x 90 m were retrieved from the NASA Shuttle Radar Topographic Mission’s (SRTM) Consortium for Spatial Information of the Consultative Group for International Agricultural Research (CGIAR-CSI) database. Soil type data at a spatial resolution of 9 x 9 km, including soil pH, bulk density, and organic carbon content, were extracted from the International Soil Reference and Information Center’s (ISRIC) World Inventory Soil Emission Potentials (WISE), version 1.0 (see; http://www.isric.org). LULC classes were merged into four categories according to similarity and respective frequencies. Yearly means, minima, and maxima of EVI, LST, and RFE were calculated for a 1-year period (May 2006 to April 2007).

### Statistical Analysis

All survey data were double-entered using EpiData version 3.1 (Epidata Association; Odense, Denmark) and validated. Environmental data processing, geo-referencing, and map drawing were made in ArcMap version 10 (ESRI; Redlands, United States of America). Environmental data were linked to parasitological and questionnaire data, by unique location. Data management and analysis of proportions and means were done in STATA version 12 (StataCorp LP; College Station, United States of America).

Two outcomes were considered in this study. First, the hookworm infection status of a participant, which was considered positive, if at least one hookworm egg was found in any of the two Kato-Katz thick smears. Second, the intensity of infection, which was calculated as follows. For each participant, the two hookworm egg counts from the duplicate Kato-Katz thick smears were summed up and multiplied by a factor 12 to obtain a standard measure of eggs per 1 g of stool (EPG). Intensity classes were created based on cut-offs put forward by the World Health Organization (WHO): light (1–1,999 EPG), moderate (2,000–3,999 EPG), and heavy (≥4,000 EPG) [24]. Age was categorized into five groups: (i) <5 years; (ii) 5–17 years, (iii) 18–34 years, (iv) 35–49 years, and (v) ≥50 years. Original categories of variables with frequencies under 5% were merged with similar categories. A socioeconomic index was built using house construction material and asset ownership, using multiple correspondence analysis (MCA) [25,26]. Households were classified in five wealth quintiles, the first quintile corresponding to the most poor and the fifth to the least poor.

The geometric mean EPG was calculated including both positive and zero counts and using the natural logarithm of the EPG augmented by 1 (ln(EPG+1)). Interactions were checked for hookworm infection prevalence and intensity, in absence of random effects in STATA, using logistic and negative binomial (NB) regression, respectively, and the likelihood ratio test (LRT). To explore the relationship between each outcome and age, smoothed age-prevalence and age-intensity curves were produced with the “mkspline” command in STATA, that regresses each outcome against a new age variable containing a restricted cubic spline of age. The same approach was used to describe the relationship between hookworm prevalence and infection intensity at village-level. Mean intensity of infection was regressed against a new prevalence variable containing restricted cubic splines of village-level prevalence, using a NB regression model.

### Model Selection for Hookworm Infection Risk

Mixed effects logistic regressions were used to model hookworm infection risk. First, to identify the best set of environmental variables to model infection risk, variable selection was performed with a Bayesian approach, the stochastic search variable selection (SSVS), using alternately non-spatial (exchangeable) or geostatistical random effects [27]. An explicit description of this method is available elsewhere [28]. Summary measures for LST, EVI, RFE, altitude, soil pH, bulk density (aimed as a proxy for soil type, a bulk density of 1.6 kg/dm^3^ corresponding to sandy soils), carbon organic content for the upper 20 cm soil layer, and land cover were fed into the aforementioned models.

Second, model validation was used to identify the model with the best predictive ability. Models including environmental covariates selected as described above were run using 10 of the 51 surveyed villages (19.6%) as test locations, while model fitting was performed on the remaining 41 villages (80.4%). To assess the predictive ability of each model, predictions for the 10 test locations were compared to observed prevalence using the mean squared error (MSE), which is the squared difference between the predicted and the observed values. The deviance information criterion (DIC) was also used to compare model fit.

### Model Selection for Hookworm Infection Intensity

Intensity of infection was approximated by the number of excreted eggs [29]. Because worm burden tends to be highly aggregated across individuals, intensity data contains many zero egg counts and are over-dispersed compared to Poisson. Count distributions accounting for data over-dispersion include negative binomial (NB), zero-inflated Poisson (ZIP), or zero-inflated negative binomial (ZINB). The NB distribution incorporates extra-Poisson variation through a dispersion parameter, r. Zero-inflated models account for over-dispersion by assuming two sources of zeros, (i) structural (non-random) zeros with probability π (mixing proportion), and (ii) zeros arising from the count distribution, i.e., Poisson (ZIP) or NB (ZINB), with probability 1 –π [30]. Further information about model specification is available in S1 Appendix.

Similarly to prevalence, variable selection was first conducted to identify the best set of environmental covariates, for spatial and non-spatial NB, ZIP, and ZINB models. Then, to identify the model with the best predictive ability, geostatistical and non-spatial NB, ZIP, and ZINB models were fitted, using the environmental sets of covariates selected for each model. Similarly as for infection risk, 41 locations were used for model fitting and the remaining 10 villages were used as test locations. Village-level means of predicted intensities at test locations obtained from all distributions were compared to the observed intensity, using the MSE. The DIC measure was also used to compare model fit.

### Bayesian Models of Hookworm Prevalence and Intensity of Infection

Three series of models were run for each outcome, using the model formulation with the best predictive ability identified by model validation. First, models without covariates using alternately a geostatistical and an exchangeable random effect were run to quantify the extent of village-level spatial correlation and unexplained variance of hookworm prevalence or intensity of infection. Second, a risk factor analysis was performed for each outcome, using demographic, socioeconomic, behavioral, and environmental determinants. Third, predictions of infection risk and intensity at non-surveyed locations were made with models using environmental covariates only.

### Risk Factor Analysis of Hookworm Infection Risk and Intensity

For each outcome, the model with the best predictive ability was used to identify the most important demographic, socioeconomic, and behavioral factors, using the SSVS procedure as described previously. Environmental variables obtained during the model selection were fed into the variable selection model together with the following questionnaire-derived variables: sex, age categories, and wealth quintiles. These variables were considered as confounders and fixed in the regression model. The same set of demographic, socioeconomic, water and sanitation, and behavioral variables was subjected to selection for each outcome and included ethnicity, education attainment, main occupation, toilet availability, raising farm animals, vegetable farming, house floor material, unsafe drinking water source, distance to drinking water source in the dry season, disposal of infant feces, wearing shoes, and drinking bottled or boiled water.

### Prediction of Hookworm Infection Risk and Intensity

Predictions at non-surveyed locations were made using models with the best predictive ability for infection risk and intensity, at a 1 x 1 km spatial resolution resulting in a 15,156-pixel grid.

### Parameter Estimation

Geostatistical models were fitted in WinBUGS version 1.4.3, using the “*spatial*.*unipred”* function (Imperial College & Medical Research Council; London, United Kingdom) [31]. A stationary isotropic process was assumed with village-specific random effects, following a multivariate normal distribution with mean zero, and a variance-covariance based on an exponential correlation function of the distance between pairs of locations. Non-informative prior distributions were chosen for all parameters. Sensitivity analysis was conducted to verify that similar results were obtained with alternate vague priors. Further information is provided in S1 Appendix. Markov chain Monte Carlo (MCMC) simulation was used to estimate model parameters [32]. For all models, two chains were run with a burn-in of 5,000. Depending on the model, variable selection was run on 100,000 to 600,000 iterations. Results were recorded for the last 1,000 iterations of each chain. For all other models, 100,000 iterations were run and results were withdrawn for the last 10,000 iterations of each chain, with a thinning of 10. Convergence was assessed through visualization of history and density plots of selected parameters. Before drawing samples, it was verified for each parameter that the Monte Carlo (MC) error, which measures the uncertainty due to simulation error, was below 5% of the standard deviation [33]. The DIC was withdrawn after 5,000 additional iterations.

## Results

### Study Population

Overall, 4,380 people were invited, among whom 280 were absent during registration, 709 missed the parasitological or the questionnaire survey, and 20 had incomplete questionnaire data. Hence, the final sample consisted of 3,371 individuals from 815 households in 51 villages.

Table 1 shows the characteristics of the 3,371 participants who had complete parasitological and questionnaire data. There were slightly more females (n = 1,776, 52.7%) than males. The mean age was 26.9 years and the inter-quartile range was 32.4 years. The age structure was as follows: <5 years (9.6%), 5–17 years (36.6%), 18–34 years (19.6%), 35–49 years (17.2%), and ≥50 years (16.9%). The illiteracy rate was 28.6% with a significant sex difference (32.6% among females and 24.2% among males; χ^2^ = 29.02, p<0.001). The most frequent occupation was rice farmer (39.7%). Over three quarters of the participants (76.3%) did not have access to sanitation of any type, and 27.4% (14/51) of villages had no toilets facilities at all. No village had toilets for all households. About one third of the study population (31.1%) did not have access to safe drinking water. About one out of five participants declared not wearing shoes outside their house.

**Table 1:** Characteristics of 3,371 study participants from Champasack province, southern Lao PDR in 2007.

Variable	Category	n (%)
Sex	Male	1,595 (47.3)
	Female	1,776 (52.7)
Age (years)	<5	325 (9.6)
	5–17	1,235 (36.6)
	18–34	661 (19.6)
	35–49	579 (17.2)
	≥50	571 (16.9)
Ethnic group	Lao Loum	2,755 (81.7)
	Other	616 (18.3)
Educational attainment	Illiterate	965 (28.6)
	Primary school	1,767 (52.4)
	Secondary school and higher	639 (19.0)
Main occupation	Rice farmer	1,339 (39.7)
	School pupil	897 (26.6)
	Tertiary sector and other	403 (12.0)
	No occupation	732 (21.7)
Socioeconomic status	Most poor	582 (17.3)
	Very poor	657 (19.5)
	Poor	739 (21.9)
	Less poor	715 (21.2)
	Least poor	678 (20.1)
Access to toilets	No	2,572 (76.3)
	Yes	799 (23.7)
Source of drinking water, dry season	Safe (village pump, protect well, pipe)	2,322 (68.9)
	Unsafe (river, pond, canal, rain)	1,049 (31.1)
Walking distance to drinking water source (min)	≤4	1,640 (48.7)
	5–9	887 (26.3)
	≥10	844 (25.0)
Consumption of bottled of boiled drinking water	No	1,518 (45.0)
	Yes	1,853 (55.0)
Does your house have a place to grow vegetables?	No	876 (26.0)
	Yes	2,495 (74.0)
Do you raise farm animals?	No	278 (8.3)
	Yes	3,093 (91.7)
House floor material	Wood	2,980 (88.4)
	Clay or bamboo	162 (4.8)
	Concrete	229 (6.8)
Disposal of infant feces	Not applicable	609 (18.1)
	Safe disposal	1,667 (49.5)
	Unsafe disposal	1,095 (32.5)
Wear shoes outside house	No	655 (19.4)
	Yes	2,716 (80.6)

### Hookworm Infection Prevalence and Intensity

**Fig 1:**
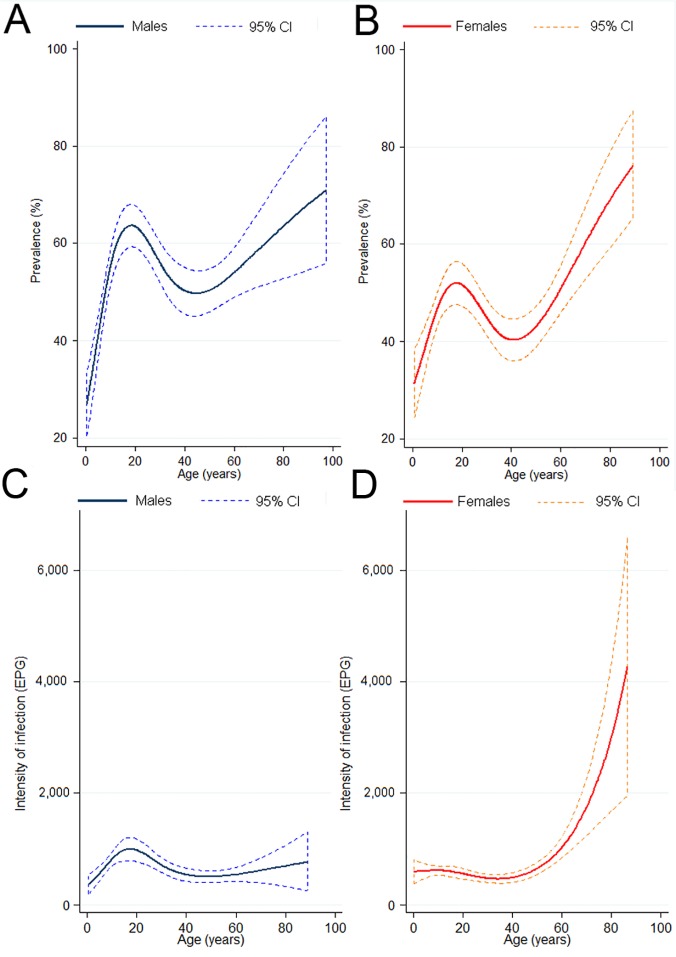
Smoothed age-prevalence and intensity curves of hookworm infection, Champasack province, southern Lao PDR. Data were obtained from a cross-sectional survey carried out among 3,371 participants in 51 villages of Champasack province in 2007. Restricted cubic splines were used. For hookworm prevalence data are stratified for males (A) and females (B). For intensity of infection, only participants with an infection were included and data are presented separately for males (C) and females (D). Uncertainty is expressed as 95% confidence interval (CI).

**Fig 2:**
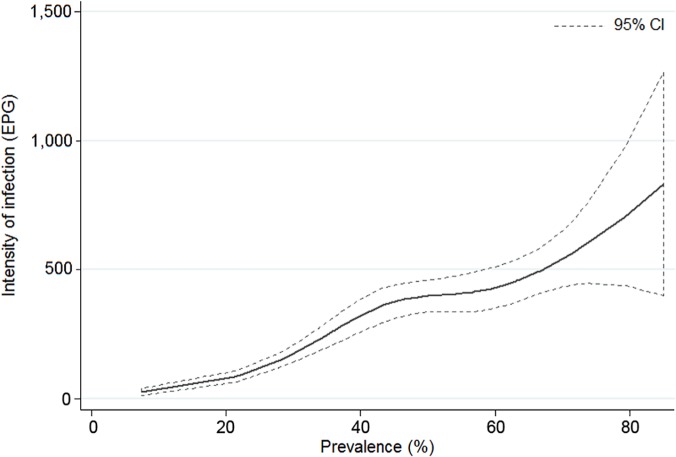
Smoothed age-intensity curve for hookworm infection intensity according to village-level prevalence, Champasack province, southern Lao PDR. Data were obtained from a cross-sectional survey carried out among 3,371 participants in 51 villages of Champasack province in 2007. The smoothed age-intensity curve is based on a restricted cubic spline of hookworm infection intensity at the unit of the village.

The overall prevalence of hookworm infection was 48.8% (95% confidence interval (CI): 47.1–50.5%). Infected individuals were found in all villages and the prevalence ranged from 7.3% (95% CI: 2.4%-16.4%) to 85.0% (95% CI: 70.2%-94.3%) at the unit of the village. Most infections (91.7%) were of light intensity according to WHO classification (1–1,999 EPG), whereas the remaining infections were either moderate or heavy (4.1% for both classes). The geometric mean intensity of infection was 12.9 EPG (95% CI: 11.6–14.3). Village-level geometric means ranged between 0.44 EPG (95% CI: 0.04–0.98) and 115.3 EPG (95% CI: 53.3–248.1). The most heavily infected individual, a 13-year-old boy, had a fecal egg count of 38,748 EPG. Smoothed age-prevalence curves, stratified by sex, are shown in Fig. 1A (males) and 1B (females). Smoothed age-infection intensity curves, for infected participants only, are displayed in Fig. 1C for males and in Fig. 1D for females. Fig. 2 displays a smoothed curve of infection intensity as a function of village-level prevalence, which were found to be positively correlated.

### Spatial Correlation of Hookworm Infection Risk and Intensity

Models run in absence of covariates indicated very little spatial correlation of hookworm infection risk and intensity. The parameters of those models are presented in Table 2. The small residual (unexplained) within-village variance (σ^2^) in the spatial model indicates a weak clustering tendency both for infection risk (σ^2^ = 0.56) and intensity (σ^2^ = 0.66). Accordingly, the spatial correlation of infection risk and intensity became less than 5% after 2.26 km and 2.04 km, respectively, indicating small spatial correlation.

**Table 2:** Parameters for the non-spatial and geostatistical logistic and NB models without covariates.

Model parameters	Prevalence				Intensity of infection			
	Non-spatial		Spatial		Non-spatial		Spatial	
	Median	95% CI	Median	95% CI	Median	95% CI	Median	95% CI
σ^2^ ^a^	0.56	0.36–0.94	0.56	0.35–0.92	0.66	0.38–1.09	0.66	0.38–1.12
ρ ^b^	n.a.	n.a.	143.5	14.5–286.4	n.a.	n.a.	158.8	27.7–287.5
Range (km) ^c^	n.a.	n.a.	2.26	1.13–22.6	n.a.	n.a.	2.04	1.12–12.64
r ^d^	n.a.	n.a.	n.a.	n.a.	0.09	0.09–0.10	0.09	0.09–0.10
DIC^e^	4,337.2	n.a.	4,336.9	n.a.	29,447.5	n.a.	29,447.1	n.a.

Parasitological data were obtained from a cross-sectional survey carried out among 3,371 participants in 51 villages of Champasack province in 2007.

CI, credible interval

Prevalence models: Bayesian geostatistical logistic model (spatial model) and Bayesian model with an exchangeable random effect (non-spatial model)

Intensity models: Bayesian geostatistical NB model (spatial model) and Bayesian NB model with an exchangeable random effect (non-spatial model)

^a^ σ^2^ is the location-specific unexplained variance

^b^ ρ is the decay parameter

^c^ The range (range = 3/ρ) is the distance at which the spatial correlation becomes less than 5%

^d^ r is the dispersion parameter from the negative binomial distribution that quantifies the amount of extra-Poisson variation

^e^ Deviance information criterion.

### Results of Model Validation

In the sensitivity analysis, all tested alternative vague priors produced similar estimates for all parameters. For infection risk, comparing the geostatistical and non-spatial logistic models, both the MSE and DIC indicated that the geostatistical (MSE = 0.0337; DIC = 4,337.2) and the non-spatial models (MSE = 0.0345; DIC = 4,336.6) had similar predictive abilities and fitted equally well. Therefore the non-spatial model was used to analyze and predict hookworm infection risk.

Among the three distributions (NB, ZIP, and ZINB) used to model hookworm infection intensity, the NB models were found to have the best predictive ability. Results of the model validation (i.e., MSE of each model), are available in S1 Table. The MSE indicated a better predictive ability for the NB geostatistical model (MSE = 36,070) than for its non-spatial counterpart (MSE = 36,826). However, model fit was similar as indicated by the DIC of the geostatistical model (DIC = 29,446.5) and the non-spatial model (DIC = 29,446.3). Although the spatial model had a 2% better predictive ability, based on the low spatial correlation of both hookworm infection risk and intensity, the similar fit, and consistency with infection risk results, the most parsimonious model, i.e. the NB model with an exchangeable random effect, was used for the analysis of infection intensity.

### Risk Factors for Hookworm Infection Risk and Intensity

The results of the mixed effects multivariate logistic and NB models, including variables selected by the SSVS procedure, for each model, are presented in Table 3. Female sex and a higher socioeconomic status (i.e., belonging to the less or least poor quintiles) were associated both with lower hookworm infection risk and intensity. With regard to age, preschool-aged children had the lowest risk. No significant interaction between age and sex was found for hookworm prevalence, but sex was an effect modifier of age for infection intensity, as indicated by a significant LRT (χ^2^ = 27.24, p<0.001). Interaction terms were accordingly included in the Bayesian mixed effects NB model. No additional important interaction was found. Preschool-aged boys harbored the lightest hookworm intensities, whilst all other age groups had similar hookworm intensities. Comparing females across age groups, women ≥50 years harbored the heaviest hookworm intensities.

**Table 3:** Determinants of prevalence and intensity of hookworm infection.

Covariate		Prevalence		Intensity of infection	
		OR	95% CI	IRR	95% CI
Sex^a^	Male	1.00		1.00	
	Female	0.75	0.65–0.86	0.56	0.39–0.82
Age (years)^b^	5–17	1.00		1.00	
	<5	0.31	0.23–0.41	0.17	0.10–0.32
	18–34	0.82	0.67–1.03	0.87	0.53–1.43
	35–49	0.77	0.62–0.96	0.64	0.40–1.05
	≥50	1.16	0.93–1.44	0.94	0.57–1.62
Interaction: effect of age among females	5–17	n.a.	n.a.	1.00	
	<5	n.a.	n.a.	0.62	0.34–1.26
	18–34	n.a.	n.a.	0.61	0.40–0.96
	35–49	n.a.	n.a.	0.85	0.51–1.40
	≥50	n.a.	n.a.	2.22	1.39–3.57
Interaction: females compared to males, among each age group	5–17	n.a.	n.a.	1.00	
	<5	n.a.	n.a.	2.02	0.93–4.44
	18–34	n.a.	n.a.	0.40	0.23–0.70
	35–49	n.a.	n.a.	0.74	0.41–1.29
	≥50	n.a.	n.a.	1.32	0.72–2.38
Ethnic group	Other	n.a.	n.a.	1.00	
	Lao Loum	n.a.	n.a.	1.58	0.98–2.72
Socioeconomic status	Most poor	1.00		1.00	
	Very poor	0.78	0.59–1.04	0.70	0.44–1.10
	Poor	0.82	0.63–1.08	0.71	0.46–1.18
	Less poor	0.64	0.49–0.85	0.48	0.29–0.77
	Least poor	0.50	0.36–0.69	0.44	0.25–0.75
Disposal of baby stools	Not applicable	1.00		1.00	
	Safe disposal	1.07	0.85–1.30	0.84	0.59–1.21
	Unsafe disposal	1.36	1.06–1.72	1.35	0.90–2.01
Source of drinking water, dry season	Safe	n.a.	n.a.	1.00	
	Unsafe	n.a.	n.a.	1.34	0.89-2.00
Distance to drinking water, dry season	≤ 4 minutes	n.a.	n.a.	1.00	
	5–9 minutes	n.a.	n.a.	0.69	0.50–0.96
	≥ 10 minutes	n.a.	n.a.	0.98	0.67–1.44
House floor material	Wood	1.00		1.00	
	Clay/bamboo	0.85	0.57–1.27	0.78	0.41–1.58
	Concrete	1.30	0.93–1.88	0.75	0.42–1.30
Raise farm animals	No	n.a.	n.a.	1.00	
	Yes	n.a.	n.a.	1.49	0.94–2.30
LST day (monthly minimum)		1.23	0.99–1.54	1.32	1.06–1.65
Soil bulk density	[1.2–1.4 [kg/dm^3^	1.00		1.00	
	[1.4–1.6 kg/dm^3^	0.50	0.30–0.81	n.a.	n.a.
**Model parameters**					
σ^2^ (median)^c^		0.51	0.32–0.81	0.57	0.32–1.01
r (median)^d^		n.a.	n.a.	0.09	0.09–0.10
DIC^e^		4,243.3	n.a.	29,376.2	n.a.

Results obtained using multivariate non-spatial models and data from a cross-sectional parasitological and questionnaire survey, Champasack province, southern Lao PDR in 2007. Prevalence models: Bayesian non-spatial mixed effects logistic model

Intensity models: Bayesian non-spatial mixed effects NB model

CI, credible interval

OR, odds ratio (posterior median)

IRR, incidence rate ratio (posterior median)

^a^ For the intensity of infection, IRRs correspond to the effect of sex among the baseline age group (5–17 years)

^b^ For the intensity of infection, IRRs correspond to the effect of age among males

^c^ σ^2^ is the location-specific unexplained variance

^d^ r is the dispersion parameter from the negative binomial (NB) distribution that quantifies the amount of extra-Poisson variation

^e^ Deviance information criterion.

### Prediction of Hookworm Infection Risk

**Fig 3:**
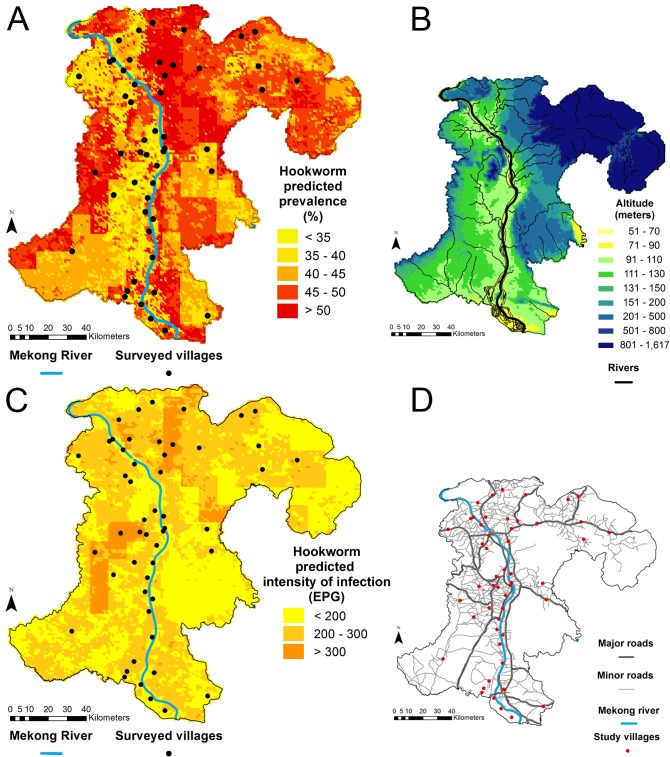
Maps of predicted hookworm prevalence (A), elevation (B), predicted hookworm infection intensity (C), and road network (D) in Champasack province, southern Lao PDR. Predictions were based on the non-spatial mixed effects logistic (prevalence) and NB (infection intensity) models using environmental covariates only.

**Fig 4:**
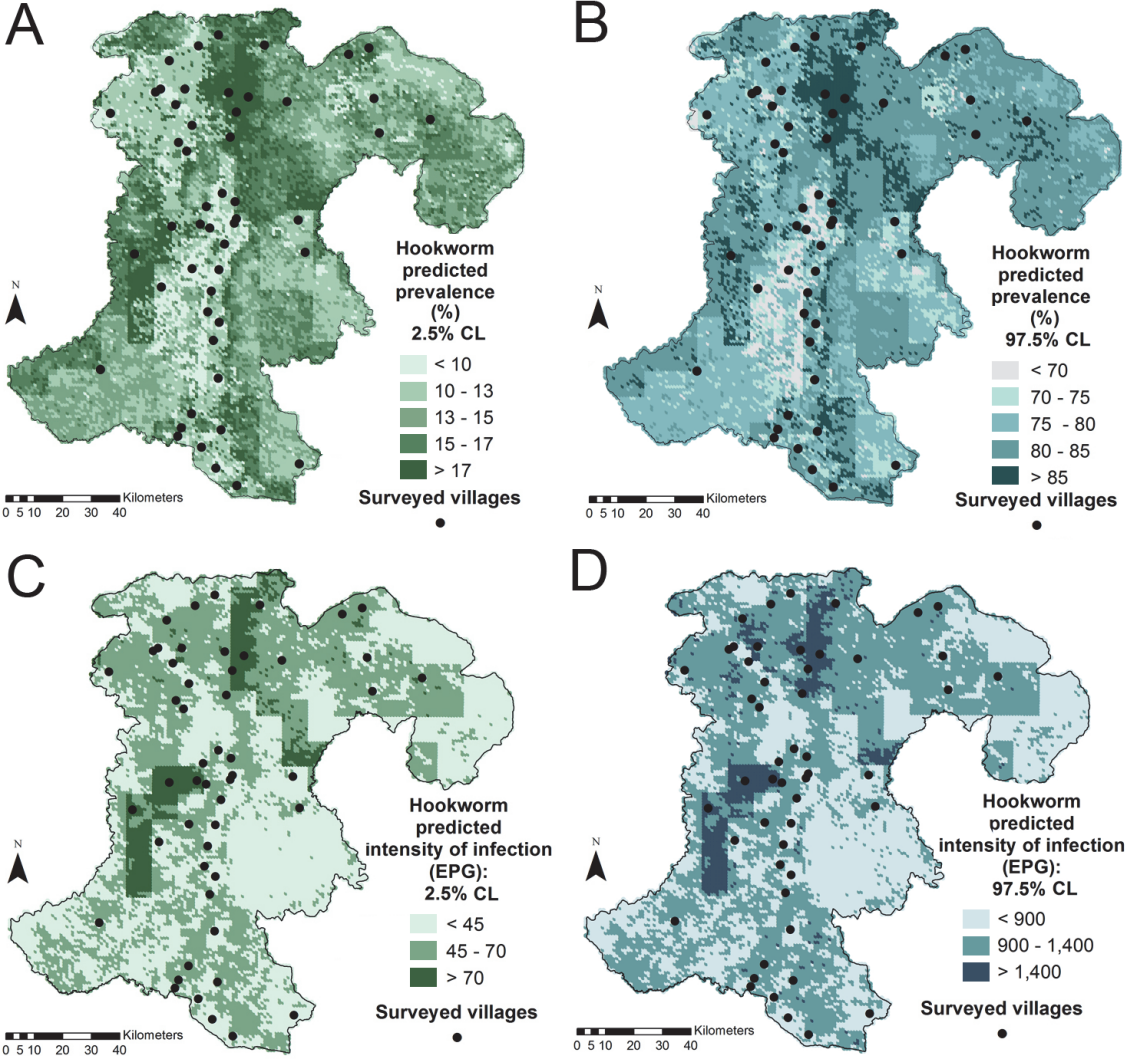
Uncertainty of hookworm predictions in Champasack province, southern Lao PDR. Lower estimates (2.5% CL) of hookworm predicted risk (A) and infection intensity (C). Upper estimates (97.5% CL) of hookworm predicted risk (B) and infection intensity (D). CL, credible limit.

Hookworm infection risk was predicted with the non-spatial logistic model, which included four covariates: LST day, land cover, soil bulk density, and organic carbon. Bayesian model fit indicated that no environmental predictor was important. ORs for this model are presented in S2 Table. Fig. 3A displays the median of the posterior predicted distribution of hookworm prevalence in Champasack province. Hookworm was found to be ubiquitous in this setting, with somewhat lower prevalence rates predicted in the lowlands, along the west side of the Mekong River in the center and southern parts of the province, and on both banks in the north-western part. Elevation is displayed in Fig. 3B. The uncertainty of predictions was large, but the geographic distribution of prevalence was maintained in the lower (2.5%) and upper (97.5%) limits of the Bayesian credible intervals. Fig. 4A and 4B display the lower and upper prediction boundaries for predicted prevalence, respectively. Maps of environmental characteristics of the province are available as supplementary information in S1 Fig.

### Prediction of Hookworm Infection Intensity

Hookworm infection intensity was predicted using the non-spatial NB model, with LST day and soil bulk density included as covariates. Higher minimum LST day was positively associated with hookworm infection intensity. IRRs for this model are available in S2 Table. The median of the posterior predicted distribution of hookworm infection intensity is presented in Fig. 3C. All estimates corresponded to light hookworm infection intensities (maximum: 599.3 EPG), with comparable levels all over the province. Slightly lighter infections were predicted closely around most survey villages located right on the Mekong River, and in areas with low population density and road network (i.e., in some zones of the south-west, in the center-east, on the borders of the north-east). Fig. 3D presents the road network of Champasack province. The uncertainty of predicted infection intensity was substantial. Still, the pattern of predicted intensity persisted in the lower (2.5%) and upper (97.5%) limits of the Bayesian credible intervals, which are shown in Fig. 4C and 4D, respectively.

## Discussion

Results from a cross-sectional parasitological and questionnaire survey reported here with more than 3,000 participating individuals from 51 villages confirm that hookworm infection is highly endemic in the Champasack province. The prevalence of hookworm was considerably higher than that of the other soil-transmitted helminths (i.e., *Ascaris lumbricoides* and *Trichuris trichiura*) [16,20,34]. Indeed, almost every second individual was infected with hookworm, while only few individuals were infected with *A*. *lumbricoides* and *T*. *trichiura*. Interestingly, hookworm infections were mainly of light intensity; 91.7% of the infections were below 2,000 EPG. We used duplicate Kato-Katz thick smears based on a single stool sample for hookworm diagnosis. Hence, the reported prevalence of infection underestimates the “true” situation, due to the low sensitivity of the Kato-Katz technique, particularly in areas where light infections predominate [35–37]. This issue is underscored by a previous study: Kato-Katz thick smears examined from two stool samples, combined with a formalin-ethyl-acetate concentration technique on a third stool sample, revealed a hookworm prevalence of 76.8% in six villages in the same province [20]. An additional concern might be the potential bias due to the 84% compliance in this study, but the missing data did not affect the age, sex, or socioeconomic composition of datasets, which were similar before and after excluding participants who did not have parasitological data.

Hookworm infections were encountered in all of the 51 villages surveyed and exhibited only little spatial clustering. This observation is in line with previous findings from Côte d’Ivoire and elsewhere in Africa [38,39].

The spatial correlation was negligible, and hence, predictions of hookworm infection risk and intensity were obtained using non-spatial models. Lower hookworm prevalence rates were predicted in the lowlands, mostly along the western side of the Mekong River. Light infection intensities were predicted all over the province, with an overall homogeneous distribution.

The current study has two main limitations that may have impacted the precision of our estimates. First, there is a lack of sampled locations in the south-west, the center-east, and the north-east mountains. While this is reflecting the lower population densities in these areas, it resulted in higher prediction uncertainty, which was particularly substantial for infection intensity. It might also explain the absence of association between hookworm prevalence and elevation, although lower risk was predicted in the lowlands, at altitudes below 150 m. We also found no association between elevation and *O*. *viverrini* infection risk in the same province, although risk zones for this parasite were clearly delineated by altitude [21]. Additionally, our models did not account for ongoing control efforts, which might have further increased uncertainty of estimates, particularly in this setting, where infection intensity is low, yielding a high sensitivity to treatment. Given the low precision of hookworm prevalence and infection intensity predictions, setting-specific estimates must be interpreted with caution. However, despite the large credible intervals of the predictions, the same patterns of predicted risk and intensity arose in the lower and upper prediction estimates, and appear to be reliable. Finally, the uncertainties we report here for both outcomes are in line with those reported by other mapping studies of hookworm, *S*. *mansoni*, and *S*. *haematobium* mono- or co-infections in different parts of Africa [38–41]. It is conceivable that our predictive risk model captured some unmeasured processes related to the province topography and associated human features, including helminthiasis control measures. The province zones with lower predicted prevalence (i.e., the lowlands), and particularly the areas bordering the Mekong River, have a higher population density, a more advanced infrastructure with a denser road network, offer better living conditions, access to health care, and benefit from a higher coverage of helminthiasis control interventions than mountains and remote areas (i.e., province South-West) [20]. Importantly though, the first round of the national deworming program targeting school- and preschool-aged children was delivered in the Champasack province in October 2006 (i.e., 3–9 months before the present survey) [9].

However, infection levels of children attending primary schools (i.e., who received a single oral dose of mebendazole (500 mg) through the deworming program), were similar to infection levels of children attending secondary schools, and who were not dewormed. This suggests either high re-infection rates, or a limited impact of the initial deworming round in 2006. Monitoring of the control program after two rounds of deworming showed disappointing results for hookworm infections, probably due to the low efficacy of mebendazole against this helminth species [9,42,43]. A recent study conducted in Champasack province found that a single oral dose of mebendazole (500 mg) resulted in a low cure rate (CR) of only 17.6% and a moderate egg reduction rate (ERR) of 76.3% against hookworm infection [34]. It must be noted, however, that our data did not allow adjusting for individual treatment. The available treatment data were self-reported, and hence are prone to reporting bias. Indeed, only 13.2% of participants and 12.0% of children attending primary school reported to ever have received deworming drugs, although the latter benefited from the national school-based deworming program which reported a 95% national coverage in 2007 [9,42].

Although no causality can be inferred given the cross-sectional design of the present survey, an interesting result was that women aged ≥50 years had heavier hookworm infections than younger females. Elder women were also found to have high hookworm infection intensities in Vietnam, Hainan province of the People’s Republic of China, and Uganda [44–46]. Although the use of night soil in agriculture could explain this result in Vietnam, this practice was absent in Hainan province, similarly to our study setting [44,45]. Interestingly, 68.1% of participants aged 50 years and above who reported no occupation outside the household were women, while 30.6% of women aged ≥50 years reported having no occupation, a proportion raising to 55.8% and 88.3% for women aged ≥60 and ≥70 years, respectively. By spending more time at home, they might be more exposed to contaminated environment at and around their place of residence. This assumption is consistent with the results of two household-based studies conducted in Uganda and Brazil, which concluded that exposure to hookworm was mostly concentrated in the peri-domiciliary environment [46,47]. However, the relative roles of exposure, immune response, and host genetic susceptibility in the epidemiology of hookworm infections are still poorly understood [44,48].

Poor hygiene, lack of sanitation, and clean water are well known to influence hookworm transmission [38,46,49,50]. However, we did not find any association between hookworm infection levels and most of water and sanitation indicators, including self-reported availability of toilets, which contradicts findings from two recent systematic reviews and meta-analyses [51,52]. No village had full sanitation coverage, and hence, soil contamination with hookworm and other helminth species occurred in all survey locations.

This lack of association might also relate to the exposure to specific helminth species such as *Ancylostoma ceylanicum*, a hookworm of dogs and cats widely distributed and highly prevalent among carnivore pets in Asia [53,54]. This hookworm species can produce patent infections among humans and recent studies suggest that its frequency and role in human infections in Southeast Asia might be largely underestimated [54–56]. Indeed, a recent study conducted in a rural Cambodian village found that 52% of participants infected with hookworm were actually harboring *A*. *ceylanicum* [57]. Although very little, the 2 km range we found both for prevalence and intensity of hookworm infection was larger than the smallest distance between two surveyed villages. This suggests that transmission occurs within and around villages rather than between them, and could relate to the distribution of defecation sites inside villages and around living places, but maybe also to zones where human cohabit with pets and semi-domesticated cats and dogs. *A*. *ceylanicum* infections in humans should be further assessed in Southeast Asia. In a first step, infection levels should be investigated at larger scales and in various settings. Subsequently, treatment efficacy and the potential impact of this zoonotic parasite on deworming programs effectiveness and sanitation-related control measures should be determined.

In view of the high prevalence of hookworm infection but the relatively low overall infection intensity, our results suggest a low morbidity associated with hookworm mono-infections in Champasack province. Yet, hookworm is co-endemic with other soil-transmitted helminths and trematodes (e.g., *O*. *viverrini* and *S*. *mekongi*) in this setting [16–21]. High prevalence rates for these intestinal helminth species, coupled with a high proportion of poly-parasitized individuals reported in 2006 suggest potential co-morbidities that should not be overlooked [20]. Multiparasitism is the rule rather than the exception in Lao PDR and elsewhere, and hence, co-morbidity must be studied in connection with co-infections [58]. Our results suggest that hookworm control efforts should be intensified in the Champasack province, particularly in mountainous areas. In the meantime, school-based deworming targeting soil-transmitted helminthiasis, opisthorchiasis, and schistosomiasis mekongi, which resumed in 2008 and 2006, respectively, has been implemented, although irregularly [20,21]. An up-to-date assessment would be needed to evaluate the impact of these programs after several years of implementation and the evolution of parasitic infections in the region, including in the most remote areas, in light of achieved coverage, target age groups, re-infection rates, and efficacy of single-oral dose of currently delivered drugs in deworming programs.

## Supporting Information

S1 Checklist STROBE checklist. (DOC)

S1 Table Results of the model validation for hookworm prevalence and intensity risk profiling. Parasitological data were obtained from a cross-sectional parasitological and questionnaire survey, Champasack province, southern Lao PDR in 2007. Shown are spatial and non-spatial hookworm prevalence models, and spatial and non-spatial NB, ZINB, and ZIP models for hookworm infection intensity, including environmental covariates only (predictive models).
(DOCX)

S2 Table Odds ratios (ORs) and incidence rate ratios (IRRs) of environmental covariates in the predictive models. Parasitological data were obtained from a cross-sectional parasitological and questionnaire survey, Champasack province, southern Lao PDR in 2007. Results obtained with the non-spatial logistic model for hookworm infection prevalence and non-spatial NB model for hookworm infection intensity.
(DOCX)

S1 Fig Distribution of environmental factors in Champasack province, southern Lao PDR. (TIF)

S1 Appendix Formulation of logistic, NB, ZIP and ZINB models for hookworm risk profiling. (DOCX)
