# Supplementary material for: Risk Profiling of Hookworm Infection and Intensity in Southern Lao People’s Democratic Republic Using Bayesian Models
Source: PLoS Negl Trop Dis. 2015 Mar 30;9(3):e0003486. doi: 10.1371/journal.pntd.0003486 (PMC4378892; doi:10.1371/journal.pntd.0003486)
Supplement: S1 Checklist — (DOC) [file pntd.0003486.s001.doc]

**Checklist S1**

STROBE Statement

|  | Item No | Recommendation |
| --- | --- | --- |
| **Title and abstract** | 1 | (*a*) Indicate the study’s design with a commonly used term in the title or the abstract |
|  |  | Abstract, section “methodology” |
|  |  | (*b*) Provide in the abstract an informative and balanced summary of what was done and what was found |
|  |  | Abstract sections “methodology” & “principal findings” |
| Introduction | | |
| Background/rationale | 2 | Explain the scientific background and rationale for the investigation being reported |
|  |  | Introduction, paragraphs 1 &2 |
| Objectives | 3 | State specific objectives, including any pre-specified hypotheses |
|  |  | Introduction, paragraph 3 |
| Methods | | |
| Study design | 4 | Present key elements of study design early in the paper |
|  |  | Section “Parasitological, Demographic, Socioeconomic, and Behavioral Data”, paragraph 1 |
| Setting | 5 | Describe the setting, locations, and relevant dates, including periods of recruitment, exposure, follow-up, and data collection |
|  |  | Section “study area”, section “Parasitological, Demographic, Socioeconomic, and Behavioral Data”, paragraph 1. |
| Participants | 6 | (*a*) Give the eligibility criteria, and the sources and methods of selection of participants |
|  |  | Section “Parasitological, Demographic, Socioeconomic, and Behavioral Data”, paragraph 1. |
| Variables | 7 | Clearly define all outcomes, exposures, predictors, potential confounders, and effect modifiers. Give diagnostic criteria, if applicable |
|  |  | Exposures, predictors: Section “ Parasitological, Demographic, Socioeconomic, and Behavioral Data”, paragraph 2; Section “Environmental data”;  Outcomes; Section « statistical analysis », paragraph 2.  Effect modifiers: Section « statistical analysis », paragraph 3.  Diagnostic techniques : Section “ Parasitological, Demographic, Socioeconomic, and Behavioral Data”, 1st paragraph |
| Data sources/ measurement | 8* | For each variable of interest, give sources of data and details of methods of assessment (measurement). Describe comparability of assessment methods if there is more than one group |
|  |  | Section “ Parasitological, Demographic, Socioeconomic, and Behavioral Data”, paragraph 2; Section “Environmental data”; |
| Bias | 9 | Describe any efforts to address potential sources of bias |
|  |  | In this study existing data are further analysed. Therefore, controlling original bias of data collection could not be addressed. |
| Study size | 10 | Explain how the study size was arrived at |
|  |  | Section “Parasitological, Demographic, Socioeconomic, and Behavioral Data”, paragraph 1. |
| Quantitative variables | 11 | Explain how quantitative variables were handled in the analyses. If applicable, describe which groupings were chosen and why |
|  |  | Section « statistical analysis », paragraph 2. |
| Statistical methods | 12 | (*a*) Describe all statistical methods, including those used to control for confounding |
|  |  | Sections “Statistical analysis”, “Model selection for hookworm infection risk”, “Model selection for hookworm infection intensity”, “prediction of hookworm infection risk & intensity”, “Parameter estimation”  Confounding: “Risk factor analysis of hookworm infection risk & intensity” |
|  |  | (*b*) Describe any methods used to examine subgroups and interactions |
|  |  | Section “Statistical analysis” |
|  |  | (*c*) Explain how missing data were addressed |
|  |  | Results, section “study population”, paragraph 1. |
|  |  | (*d*) If applicable, describe analytical methods taking account of sampling strategy  Not applicable |
|  |  | (*e*) Describe any sensitivity analyses  Section “Parameter estimation”, paragraph 1. |
| Results | | |
| Participants | 13* | (a) Report numbers of individuals at each stage of study—eg numbers potentially eligible, examined for eligibility, confirmed eligible, included in the study, completing follow-up, and analysed |
|  |  | Results, section “study population”, paragraph 1. |
|  |  | (b) Give reasons for non-participation at each stage |
|  |  | Results, section “study population”, paragraph 1. |
|  |  | (c) Consider use of a flow diagram |
|  |  | Not done |
| Descriptive data | 14* | (a) Give characteristics of study participants (eg demographic, clinical, social) and information on exposures and potential confounders |
|  |  | Section “Study population”, paragraph 2 |
|  |  | (b) Indicate number of participants with missing data for each variable of interest |
|  |  | Not done since those individuals were dropped. |
| Outcome data | 15* | Report numbers of outcome events or summary measures |
|  |  | Section “Hookworm infection Prevalence and Intensity” |
| Main results | 16 | (*a*) Give unadjusted estimates and, if applicable, confounder-adjusted estimates and their precision (eg, 95% confidence interval). Make clear which confounders were adjusted for and why they were included |
|  |  | Table 3, Table S2 |
|  |  | (*b*) Report category boundaries when continuous variables were categorized |
|  |  | Section “Statistical analysis” paragraph 2  Table1, Table 3 |
|  |  | (*c*) If relevant, consider translating estimates of relative risk into absolute risk for a meaningful time period |
|  |  | Not applicable |
| Other analyses | 17 | Report other analyses done—eg analyses of subgroups and interactions, and sensitivity analyses |
|  |  | Interactions: Section “Risk factors for Hookworm Infection Risk and Intensity”  Sensitivity analysis: Section « Result of model validation », paragraph 1. |
| Discussion | | |
| Key results | 18 | Summarise key results with reference to study objectives |
|  |  | Paragraphs 1, 2 |
| Limitations | 19 | Discuss limitations of the study, taking into account sources of potential bias or imprecision. Discuss both direction and magnitude of any potential bias  Paragraphs 2, 3 |
|  |  |  |
| Interpretation | 20 | Give a cautious overall interpretation of results considering objectives, limitations, multiplicity of analyses, results from similar studies, and other relevant evidence  Paragraphs 1 to 8. |
|  |  |  |
| Generalisability | 21 | Discuss the generalisability (external validity) of the study results |
|  |  | Not applicable |
| Other information | | |
| Funding | 22 | Give the source of funding and the role of the funders for the present study and, if applicable, for the original study on which the present article is based |
|  |  | Study funded by the Swiss National Science Foundation |

*Give information separately for exposed and unexposed groups.

**Note:** An Explanation and Elaboration article discusses each checklist item and gives methodological background and published examples of transparent reporting. The STROBE checklist is best used in conjunction with this article (freely available on the Web sites of PLoS Medicine at http://www.plosmedicine.org/, Annals of Internal Medicine at http://www.annals.org/, and Epidemiology at http://www.epidem.com/). Information on the STROBE Initiative is available at www.strobe-statement.org.
