# Supplementary material for: Risk Profiling of Hookworm Infection and Intensity in Southern Lao People’s Democratic Republic Using Bayesian Models
Source: PLoS Negl Trop Dis. 2015 Mar 30;9(3):e0003486. doi: 10.1371/journal.pntd.0003486 (PMC4378892; doi:10.1371/journal.pntd.0003486)
Supplement: S1 Table — Parasitological data were obtained from a cross-sectional parasitological and questionnaire survey, Champasack province, southern Lao PDR in 2007. Shown are spatial and non-spatial hookworm prevalence models, and spatial and non-spatial NB, ZINB, and ZIP models for hookworm infection intensity, including environmental covariates only (predictive models). (DOCX) [file pntd.0003486.s002.docx]

**Table S1. Results of the model validation for hookworm prevalence and intensity risk profiling.**

| **Prevalence** | MSE^a^ |
| --- | --- |
| non-spatial | 0.0345 |
| spatial | 0.0337 |
| **Intensity of infection** |  |
| NB non-spatial | 36,826.47 |
| NB spatial | 36,069.89 |
| ZINB non-spatial | 38,258.58 |
| ZINB spatial | 40,069.97 |
| ZIP non-spatial | 46,303.53 |
| ZIP spatial | 38,645.35 |

Parasitological data were obtained from a cross-sectional parasitological and questionnaire survey, Champasack province, southern Lao PDR in 2007. Shown are spatial and non-spatial hookworm prevalence models, and spatial and non-spatial NB, ZINB, and ZIP models for hookworm infection intensity, including environmental covariates only (predictive models).

^a^(a) (dian) (b)catedon Criterion. a thod, cifically selected for each variant of the models (i.e. ls for infection intensity. Mean Squared error;

A lower MSE indicates a better predictive ability;

Included environmental covariates were specifically selected with the SSVS selection variable method, for each variant of the models (i.e. non-spatial *vs.* spatial), and for each distribution.
