# Supplementary material for: Risk Profiling of Hookworm Infection and Intensity in Southern Lao People’s Democratic Republic Using Bayesian Models
Source: PLoS Negl Trop Dis. 2015 Mar 30;9(3):e0003486. doi: 10.1371/journal.pntd.0003486 (PMC4378892; doi:10.1371/journal.pntd.0003486)
Supplement: S2 Table — Parasitological data were obtained from a cross-sectional parasitological and questionnaire survey, Champasack province, southern Lao PDR in 2007. Results obtained with the non-spatial logistic model for hookworm infection prevalence and non-spatial NB model for hookworm infection intensity. (DOCX) [file pntd.0003486.s003.docx]

**Table S2. Odds ratios (ORs) and incidence rate ratios (IRRs) of environmental covariates in the predictive models.**

|  |  | **Prevalence** | | **Intensity of infection** | |
| --- | --- | --- | --- | --- | --- |
| **Covariate** |  | OR | 95% CI | IRR | 95% CI |
| LST day (monthly minimum) |  | 1.19 | 0.97 - 1.47 | 1.31 | 1.07 - 1.67 |
| Soil bulk density | 1.20 - 1.39 kg/dm^3^ | 1.00 |  | 1.00 |  |
|  | 1.40 - 1.59 kg/dm^3^ | 0.63 | 0.31 - 1.35 | 0.60 | 0.35 - 1.09 |
| Soil organic carbon content | 5.00 – 9.99 g/kg | 1.00 |  | n.a. | n.a. |
|  | 10.00 – 19.99 g/kg | 0.72 | 0.42 - 1.31 | n.a. | n.a. |
| Land Cover | Savannah, grassland, shrubland | 1.00 |  | n.a. | n.a. |
|  | Water and wetlands | 0.87 | 0.47 - 1.61 | n.a. | n.a. |
|  | Forest | 1.05 | 0.48 - 2.40 | n.a. | n.a. |
|  | Cropland, bare and built soil | 0.59 | 0.34 - 1.06 | n.a. | n.a. |
| **Model parameters** |  |  |  |  |  |
| σ^2^ (median) ^a^ |  | 0.50 | 0.31 - 0.83 | 0.52 | 0.27 - 0.92 |
| r (median) ^b^ |  | n.a. | n.a. | 0.09 | 0.09 - 0.10 |
| DIC ^c^ |  | 4,336.59 | n.a. | 29,446.30 | n.a. |

Parasitological data were obtained from a cross-sectional parasitological and questionnaire survey, Champasack province, southern Lao PDR in 2007. Results obtained with the non-spatial logistic model for hookworm infection prevalence and non-spatial NB model for hookworm infection intensity.

OR, Odds Ratio (posterior median);

CI, Credible Interval;

IRR, Incidence Rate Ratio (posterior median);

^a^ σ^2^ is the location-specific unexplained variance;

^b^ r is the dispersion parameter from the Negative Binomial distribution that quantifies the amount of extra-Poisson variation;

^c^ Deviation Information Criterion.
