# Supplementary figures and images for: Risk Profiling of Hookworm Infection and Intensity in Southern Lao People’s Democratic Republic Using Bayesian Models
Source: PLoS Negl Trop Dis. 2015 Mar 30;9(3):e0003486. doi: 10.1371/journal.pntd.0003486 (PMC4378892; doi:10.1371/journal.pntd.0003486)

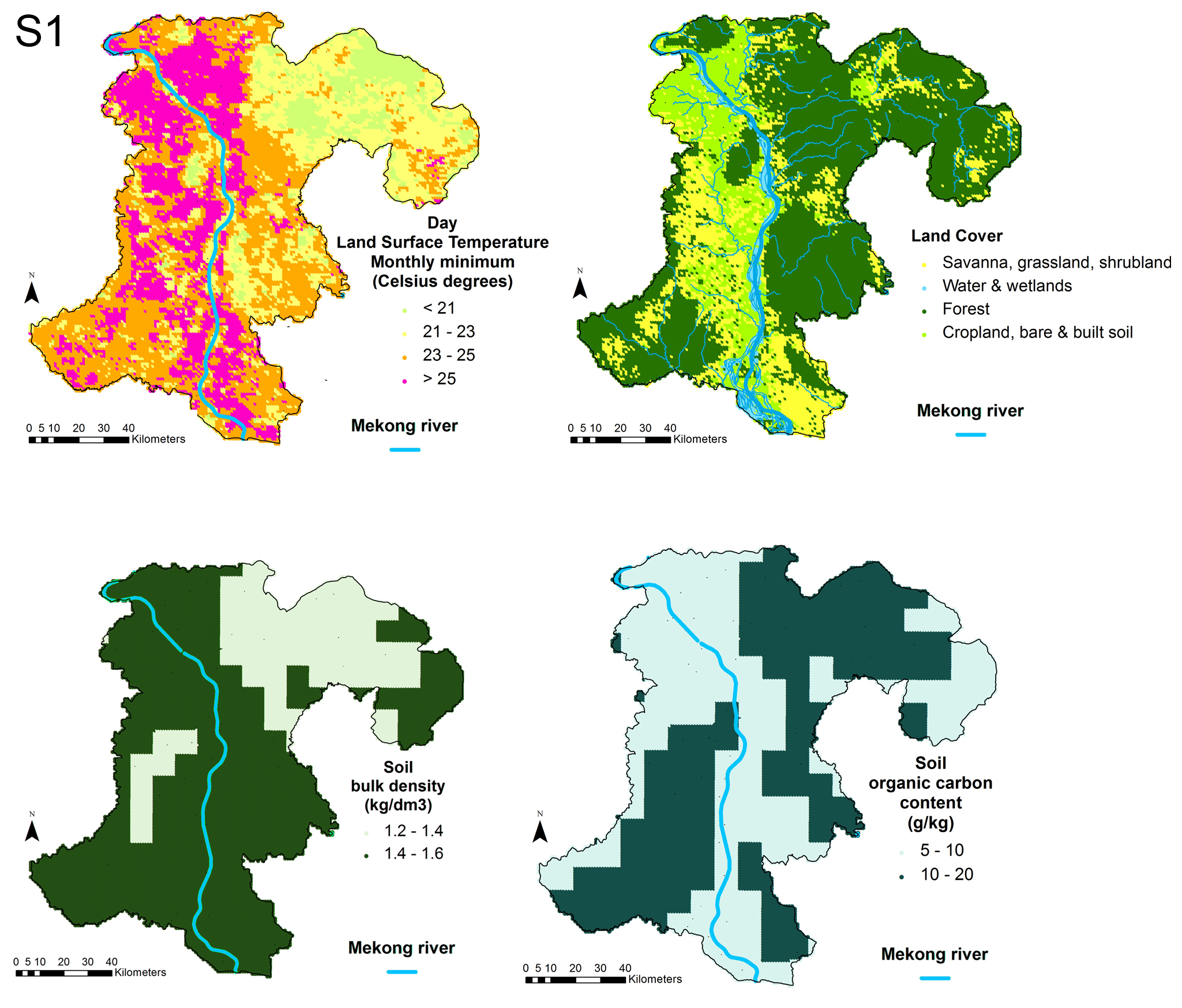

Supplement: S1 Fig — (TIF) [file pntd.0003486.s004.tif]
