# Supplementary material for: Risk Profiling of Hookworm Infection and Intensity in Southern Lao People’s Democratic Republic Using Bayesian Models
Source: PLoS Negl Trop Dis. 2015 Mar 30;9(3):e0003486. doi: 10.1371/journal.pntd.0003486 (PMC4378892; doi:10.1371/journal.pntd.0003486)
Supplement: S1 Appendix — (DOCX) [file pntd.0003486.s005.docx]

**Appendix S1: formulation of logistic, NB, ZIP and ZINB models**

Contents:

1. Analysis of hookworm infection risk: logistic model
2. Analysis of hookworm infection intensity
   1. NB model
   2. ZIP and ZINB models
3. Specifications of prior distributions
   1. Random effects
   2. Other priors
4. Sensitivity analysis
5. **Analysis of hookworm infection risk: logistic model**

Let Y*_ij_* be the infection status of individual *i* in village *j*, taking the values 0 or 1 for a negative or positive status, respectively. For an individual *i* in village *j*, we assume that Y*_ij_* ~ Bernouilli(p*_ij_*) where *p_ij_* is the probability of infection, and model *p_ij_* as follows: $\log it\left(p_{ij}\right)=\alpha+\sum_{k=1}^{n} \beta_{k}X_{kij}+\varphi_{j}$, where *X_kij_* is the k^th^, n=1,….n, covariate for individual *i* in village *j*, and $\phi_{j}$ is the locational random effect for village *j* (see section 3.1).

1. **Analysis of hookworm infection intensity: NB, ZIP and ZINB models**

**2.1 Negative binomial (NB) model**

Let Y*_ij_* be the eggs count of individual *i* in village *j*. We assume that $Y_{ij} \sim NB(p_{ij} , r)$ and model the mean eggs count λ*_ij_*, as follows:

$p_{ij}=\frac{r}{\left( r+ \lambda_{ij} \right)}$ , log($\lambda_{ij})$ = α +$\sum_{k=1}^{n} \beta_{k}X_{kij}+\phi_{j}$, where *X_kij_* is the n^th^ explanatory variable, and $\phi_{j}$ is the locational random effect for village (see section 3.1). Under this parameterization, the NB is a Poisson-Gamma mixture, where *r* is a real number that has the meaning of a dispersion parameter [1].

**2.2 Zero-inflated Poisson (ZIP) and zero-inflated negative binomial (ZINB) models**

Zero-inflated models assume that a proportion π (mixing proportion) of zeros are structural (non-random) and that the remaining zeros arise from the count distribution (Poisson (i.e. ZIP) or Negative binomial (i.e. ZINB)), with probability 1 – π. We assume that the mixing proportion π is constant across individuals and introduce covariates only on the mean parameter of the count.

Let Y*_ij_* be the eggs count of individual *i* in village *j*. We assume $Y_{ij} \sim Poisson({mu}_{ij})$,

${mu}_{ij}= \lambda_{ij}*(1-u_{ij})$ (ZIP distribution) and $Y_{ij} \sim NB(p_{ij} , r)$, $p_{ij}=\frac{r}{\left( r+ \lambda_{ij} \right)*(1- u_{ij})}$ (ZINB distribution). We model log($\lambda_{ij})$ = α +$\sum_{k=1}^{n} \beta_{k}X_{kij}+\phi_{j}$, $u_{ij}\sim Be(\pi)$, where *π* is the mixing proportion (probability of structural zeros of the inflation model) and $u_{ij}$ is a binary indicator that specifies whether the zeros are structural or arise from the count distribution.

1. **Specification of prior distributions**

**3.1 Random effects**

For all models (logistic, NB, ZIP and ZINB), we consider $\boldsymbol{\phi}={(\phi_{1}, \ldots, \phi_{51})}^{T}$ to be either an exchangeable random effect (non-spatial models) or a geostatistical random effect (spatial models). In particular, we assume *ϕ* ~ N(0 , σ^2^R*_ij_*) where σ^2^ is the variance parameter and R*_ij._*is the correlation matrix between locations. Under an exchangeable prior distribution R*_ij_* = 0 if i ≠ *j* and R*_ij_* = 1 if *i* = *j.* Under the assumption of a spatial stationary isotropic process,$R_{ij}=exp(d_{ij}, \rho)$, where *d_ij_* is the Euclidean distance between two locations *s_i_* and *s_j_*, and *ρ* is a measure of how spatial correlation decreases with the distance. The distance at which the spatial correlation between villages gets under 5% is equal to 3/*ρ* and is called the range.

A vague inverse gamma prior with mean 1 and variance 100 was chosen for σ^2^, and a uniform prior for *ρ* with parameters calculated as a function of the minimum and maximum distance between sampled villages was adopted, that is:

$\rho\sim dunif(-\frac{\log(0.01)}{\min\text{distance}};-\frac{\log(0.01)}{\max\text{distance}})$.

**3.2 Other priors**

We chose a vague Normal distribution with a mean of zero and a variance of 1000 for all the regression coefficients. The prior for the dispersion parameter *r* of the NB distribution was chosen to be a gamma with mean 1, variance 100 and a restricted domain within the range (0.001, 100). The mixing probability π of the ZIP and ZINB distributions was assumed to follow a flat beta distribution Beta(1,1).

1. **Sensitivity analysis**

For π, the mixing proportion of ZIP and ZINB models, two additional beta distributions were tested: Beta(0.5,0.5) and Beta(0.125,0.125), with mean 0.5 and variances 0.125 and 0.2, respectively. For the village-level inverse variance tau parameter (logistic, NB, ZIP and ZINB models) and the over-dispersion r parameter of the NB and ZINB models, gamma distributions with mean 1 and variances 10, 100 and 1000 were tested.

**References**

1. Ntzoufras I (2009) Bayesian Modeling Using WinBUGS: John Wiley & Sons, Inc., Hoboken, NJ, USA.
